# *In vivo* magnetic resonance imaging tracking of transplanted superparamagnetic iron oxide-labeled bone marrow mesenchymal stem cells in rats with myocardial infarction

**DOI:** 10.3892/mmr.2014.2649

**Published:** 2014-10-15

**Authors:** PING HUA, YOU-YU WANG, LI-BAO LIU, JIA-LIANG LIU, JIAN-YANG LIU, YAN-QI YANG, SONG-RAN YANG

**Affiliations:** 1 Department of Cardiothoracic Surgery, The Sun Yat-Sen Memorial Hospital, Sun Yat-Sen University, Guangzhou, Guangdong 510120, P.R. China; 2 Department of Thoracic Surgery, Sichuan Provincial People’s Hospital, Chengdu, Sichuan 610072, P.R. China; 3 Department of Neurology, Guangzhou First Municipal People’s Hospital, Guangzhou, Guangdong 510180, P.R. China

**Keywords:** superparamagnetic iron oxide, bone marrow mesenchymal stem cells, myocardial infarction, magentic resonance imaging

## Abstract

Superparamagnetic iron oxide (SPIO) nanoparticles generate superparamagnetism, thereby resulting in an inhomogeneous local magnetic field, which shortens the T2 value on magnetic resonance imaging (MRI). The purpose of the present study was to use MRI to track bone marrow mesenchymal stem cells (BMSCs) labeled with SPIO in a rat model of myocardial infarction. The BMSCs were isolated from rats and labeled with SPIO. The anterior descending branch of the coronary artery was ligated under anesthesia. Two weeks later, the rats received, at random, 5×10^7^ SPIO-labeled BMSCs, 5×10^7^ unlabeled BMSCs or a vehicle (100 μl phosphate-buffered saline) via direct injection into the ischemic area (20 animals/group). MRI was used to track the SPIO-labeled BMSCs and the rats were then sacrificed to verify the presence of BMSCs using immunohistochemistry with an anti-CD90 antibody. The procedure labeled 99% of the BMSCs with SPIO, which exhibited low-intensity signals on T2 and T2* MRI imaging. At 24 h post-BMSC transplantation, low-intensity MRI signals were detected on the T2 and T2* sequences at the infarction margins. After 3 weeks following transplantation, low-intensity signals started to appear within the infarcted area; however, the signal intensity subsequently decreased and became indistinct. Immunohistochemistry revealed that the SPIO-labeled BMSCs migrated from the margin into the infarcted region. In conclusion, the BMSCs were readily labeled with SPIO and *in vivo and* MRI tracking demonstrated that the SPIO-labeled BMSCs established and grew in the infarcted myocardium.

## Introduction

Bone marrow mesenchymal stem cells (BMSCs) are multipotent stem cells, which are able to differentiate into a variety of cell types, including myocardiocytes (1). There is increasing evidence to suggest that stem cells exist in the adult mammalian heart (2); however, when a substantial loss of cardiomyocytes occurs, including in a myocardial infarction, the endogenous cardiac regenerative mechanisms are insufficient to replace the lost cardiomyocytes. BMSCs differentiate into myocardial cells in a suitable *in vitro* microenvironment (3). In addition, BMSCs can differentiate into cardiomyocytes to replace infarcted myocardium *in vivo* (1,4), thereby improving the cardiac function that has been compromised by repeated ischemic episodes.

The assessment of the efficacy of stem cell transplantation requires an effective method to track the migration, proliferation and differentiation of the transplanted stem cells. The development of molecular imaging techniques has enabled the visualization of stem cell homing, migration, location and proliferation (5–8). Magnetic resonance imaging (MRI) possesses high temporal and spatial resolution, is noninvasive and is free of ionizing radiation. MRI can be used to observe the dynamic process of cell migration and may be a desirable approach for the *in vivo* tracking of stem cells injected with contrast agents (9–16).

Superparamagnetic iron oxide (SPIO) nanoparticles, comprised of single or multiple iron oxide crystals, are synthesized by the introduction of amine groups onto the surface of silica-coated composite magnetite nanoparticles (11). They generate superparamagnetism, thereby resulting in an inhomogeneous local magnetic field, which shortens the T2 value on MRI (17). By contrast, tissues that do not contain SPIO may show high signals on T2 and T2* sequences. Therefore, MRI using SPIO may be effective for tracking transplanted BMSCs.

SPIO nanoparticles have been used to image the movement of adipogenic mesenchymal stem cells to the infarcted myocardium (18,19). *In vivo* MRI stem cell tracking has been applied in animal models of cerebral ischemia, spinal cord injury, myocardial infarction and peripheral nerve injury (9–16). However, there have been few MRI studies on repair and functional improvement in animal models of myocardial infarction (14,16,18).

In the present study, BMSCs were labeled with SPIO and MRI was used to observe their distribution and migration following transplantation in a rat model of acute myocardial infarction.

## Materials and methods

### Animals

A total of 65 female Sprague-Dawley rats, obtained from the Laboratory Animal Center of Sun Yat-Sen University, Guangzhou, China) were used in the present study. The BMSCs were sourced from five young rats (5–6-weeks old) weighing ~150 g and the remaining 60 rats (6–8 weeks old; 180–200 g) were used in the experiments involving myocardial infarction. The rats were housed at 22°C with a 12 h light/dark cycle. Food and water were available *ad libitum*. The study procedure was approved by the Institutional Review Board of Sun Yat-Sen University and all animal experiments were performed in accordance with the established guidelines of the Institutional Animal Care and Use Committee of Sun Yat-Sen University.

### Isolation, culture and purification of BMSCs

The five young rats were anesthetized with 1.5% intraperitoneal isoflurane (30 mg/kg), sacrificed by cervical dislocation and primary BMSCs were collected, as previously described (1). Briefly, under aseptic conditions, the epiphyseal regions of the femora and tibia were removed and marrow plugs were flushed out using Dulbecco’s modified Eagle’s medium (DMEM; Gibco-BRL, Gaithersburg, MD, USA) containing 10% (v/v) fetal bovine serum (FBS; Gibco-BRL). A suspension of single bone marrow cells was obtained by repeated aspiration. The cells were seeded into 25-ml culture flasks at a density of 6×10^4^ cells/ml and were cultured in DMEM/F12 (Gibco-BRL) supplemented with 10% (v/v) FBS, 100 U/ml penicillin (Sigma-Aldrich, St. Louis, MO, USA) and 100 ng/ml streptomycin (Sigma-Aldrich) in a humidified incubator with 5% CO_2_ at 37°C. After 2 days, the culture medium and non-adherent cells were removed. The harvested cells were verified as BMSCs with flow cytometry using FITC-conjugated rat anti-mouse CD34, CD45, CD29 and CD90 monoclonal antibodies (Santa Cruz Biotechnology, Inc., Santa Cruz, CA USA). An isotope antibody was used as the negative control. The isotype antibody represented the antibody with the same sources and subgroup with FITC-conjugated rat anti-mouse CD34, CD45, CD29 and CD90 monoclonal antibodies (Santa Cruz Biotechnology, Inc.). Cells in passages 3–6 cells were used in the subsequent experiments.

### SPIO labeling of BMSCs

The SPIO (Fe^3+^_2_O_3_M^2+^O; Soochow University, Suzhou, China) used in the present study comprised a 3-aminopropyl triethoxysilane-modified Fe_2_O_3_ particle, with a diameter of 10–15 nm. SPIO labeling of the BMSCs was performed as previously described (20). Briefly, the BMSCs were washed three times with phosphate-buffered saline (PBS) and then grown in DMEM/F12 containing 10% FBS, 100 U/ml penicillin and 100 ng/ml streptomycin. The cells were then labeled with SPIO by culturing for 24 h at 37°C in an atmosphere of 5% CO_2_; the iron concentration of the culture medium was 25 μg/ml. After 24 h, the cells were washed thoroughly to remove the remaining SPIO. A total of 5×10^5^ cells were harvested for MRI detection and unlabeled BMSCs were used as the control. At 24 h after labeling, the cells were subjected to Prussian blue iron (Beijing Leagene Biotech, Co., Ltd., Beijing, China) staining using a Mallory’s method, which was performed according to standard protocols (21) and images were captured using an inverted microscope (DMi1; Leica Microsystems GmbH, Wetzlar, Germany). At least 700 cells were counted. The experiments were repeated five times independently.

### Cell viability assays

Cells (5×10^5^) were incubated with 0.4% trypan blue dye for 15 min at room temperature prior to microscopic examination. Images of four randomly selected fields were captured for each sample. The percentage of viable cells was calculated using the following formula: Cell survival rate (%) = (total number of cells - number of dyed cells)/total number of cells × 100.

For the 3-(4,5-dimethylthiazol-2-yl)-2,5-diphenyltetrazolium bromide (MTT) reduction assay, the cells were plated in a 96-well plate (1×10^4^ cells/well) and incubated in a humidified atmosphere with 5% CO_2_ at 37°C for 1–5 days prior to the MTT assay (Sigma-Aldrich). Absorbance was measured at 492 nm using a microtiter plate reader (Wellscan K3; KHB Labsystems, Helsinki, Finland).

### Acute myocardial infarction in rats

The rats were anesthetized using pentobarbital sodium (30 mg/kg, intraperitoneally), fixed on an operation table and mechanically ventilated. The ventilator maintained a breathing ratio of 1:2 at a frequency of 100 times/min and a tidal volume of 14 ml/kg. An electrocardiogram, using the standard II leads from the RM 6240 BD Multi-channel physiological signal processing system (Shanghai Huayan Instrument & Equipment Co., Ltd., Shanghai, China) was used to monitor cardiac function. From the left parasternal side, a 1.5–2.0-cm longitudinal incision was made between the third and fourth ribs and the heart was exposed following thoracotomy. The anterior descending branch of the coronary artery was ligated 2–3 mm below the left atrial appendage. The procedure was considered successful when the electrocardiogram revealed depression of the QRS-wave peak, elevation of the J point and ST-segment elevation >0.2 mV.

### Injection of BMSCs into the infarcted rat myocardium

The chest was opened under anesthesia (1.5% intraperitoneal isoflurane; 30 mg/kg) 2 weeks after coronary artery ligation and the infarcted myocardial region was fully exposed. The rats were randomly divided into three groups (20/group) and ~5×10^7^ SPIO-labeled BMSCs, an equal quantity of unlabeled BMSCs or the vehicle (100 μl PBS) was injected into the myocardial tissues along the margins of the infarcted region, which was identified by a pale color. The mean weights of the animals in the three groups were similar.

### In vivo MRI tracking of SPIO-labeled BMSCs

MRI was performed using a Philips Gyroscan Intera 1.5T Superconducting MRI scanner (Philips Medical Systems, Best, The Netherlands), with a circular surface coil of 5 cm in diameter. T2 and T2* scans were performed in transverse and oblique sagittal positions 1 day and 3 weeks after transplantation of the BMSCs. The scanning parameters for T2 were: repetition time (TR)/echo time (TE), 2,000/100 msec; slice thickness, 2.0 mm; field of view (FOV), 48×22 mm; matrix size, 256×256; number of signal averages (NSA), 2 and flip angle, 22°. The parameters for T2* were: TR/TE, 253/14 msec; slice thickness, 2.0 mm; interslice gap, 0.2 mm; FOV, 48×23 mm; matrix size, 256×256 and NSA, 3.

### Histopathological examination

Following sacrifice, the myocardial tissues were collected using the MRI images for guidance. The samples were fixed in 10% formaldehyde for 24 h and then dehydrated in gradient ethanol (100, 95 amd 75%). The samples were made transparent by soaking in xylene, then embedded in paraffin and cut into 4-μm sections. The tissue sections were first stained with hematoxylin and eosin (H&E) and were then stained using a rat anti-mouse monoclonal CD90 antibody (Santa Cruz Biotechnology, Inc.). The tissue sections were examined independently by two experienced pathologists.

### Statistical analysis

Quantitative data is expressed as the mean ± standard deviation. The differences between the SPIO-label and unlabeled BMSCs were compared using an unpaired two-sample t-test. Statistical analyses were performed using SPSS statistical software, version 15.0 (SPSS, Inc., Chicago, IL, USA). P<0.05 indicated a statistically significant difference.

## Results

### BMSC isolation

**Figure 1:**
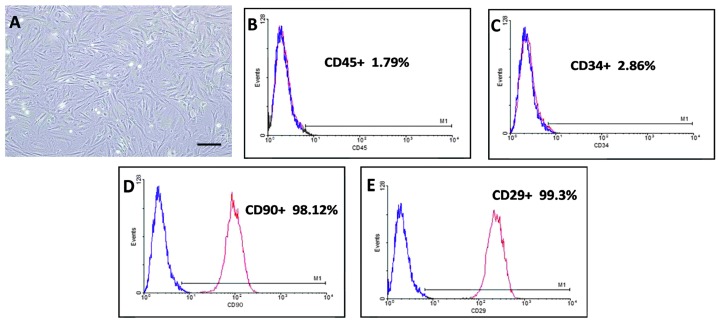
(A) Representative micrograph of the fifth generation of BMSCs of Sprague-Dawley rats, which revealed consistently long spindle-shaped cells arranged in a radial or spiral shape. These cells demonstrated colony growth under phase contrast microscopy (magnification, ×200). The expression levels of (B) CD45, (C) CD34, (D) CD90 and (E) CD29 in rat BMSCs were examined using flow cytometry. An isotype antibody was used as a negative control (blue line). The scale bar is 50 μm. BMSCs, bone marrow mesenchymal stem cells.

In the present study BMSCs were isolated from the epiphyseal regions of the femur and tibia of rats and grown *ex vivo*. The cells became adherent 6–10 h post-seeding in the culture bottle, appearing round or polygonal in shape. A few, short, spindle-like and star-like cells with pseudopodia appeared 2–3 days later. At day 7 post-seeding, radially arranged colonies were observed; these cells exhibited uneven projections, had a large nucleus and an apparent nucleolus. At 12–14 days, the cells were swirl-shaped and between 80 and 90% confluent. The cells at passages 4 and 5 demonstrated similar spindle and star-like morphological characteristics (Fig. 1A). The hematopoietic stem cell markers CD45 (Fig. 1B) and CD34 (Fig. 1C) were positive in 1.79 and 2.86% of the cells, respectively. The surface markers CD90 (Fig. 1D) and CD29 (Fig. 1E) were positive in 98.12 and 99.3% of the cells, respectively.

### SPIO labeling of BMSCs

**Figure 2:**
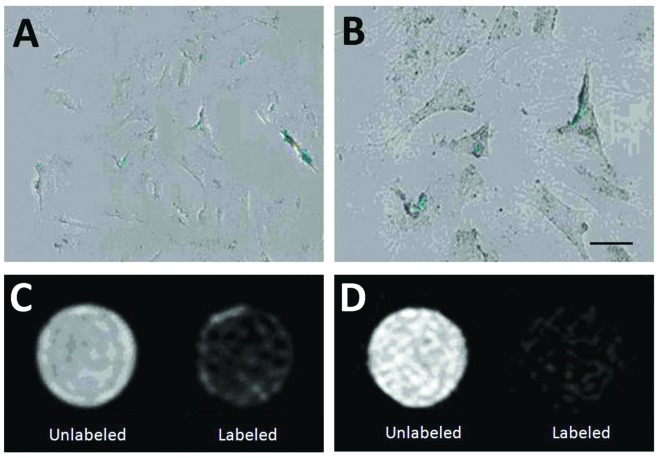
Prussian blue staining of the SPIO-labeled rat BMSCs. The labeling efficiency of the blue-stained iron particles in the cytoplasm was 99%. (A) Magnification, ×200; (B) magnification, ×400. (C) T2 and (D) T2* magnetic resonance imaging of the SPIO-labeled rat BMSCs. The scale bar is 10 μm. SPIO, superparamagnetic iron oxide; BMSCs, bone marrow mesenchymal stem cells.

Phase contrast microscopy revealed the presence of blue iron particles in 99% of the cell cytoplasm in the SPIO-labeled BMSCs (Fig. 2A and B). Compared with the unlabeled BMSCs, the SPIO-labeled cells exhibited a markedly decreased signal on the T2 and T2* sequences (Fig. 2C and D).

### SPIO exerts no immediate toxic effects on BMSCs

The viability of the SPIO-labeled and unlabeled BMSCs, examined using the trypan blue exclusion assay, are shown in Table I. No significant differences were observed in viability between the SPIO-labeled and unlabeled cells at any time point. The viabilities of the SPIO-labeled compared with those of the unlabeled cells were: 98.65±1.63 vs. 98.74±1.42 (P=0.897) after 6 h, 96.35±0.96 vs. 96.42±1.33 (P=0.894) after 12 h and 95.51±1.44 vs. 95.78±1.21 (P=0.655) after 24 h.

The results of the MTT assay indicated that SPIO did not exhibit any antiproliferative effect (data not shown).

### In vivo tracking of SPIO-labeled BMSCs by MRI

**Figure 3:**
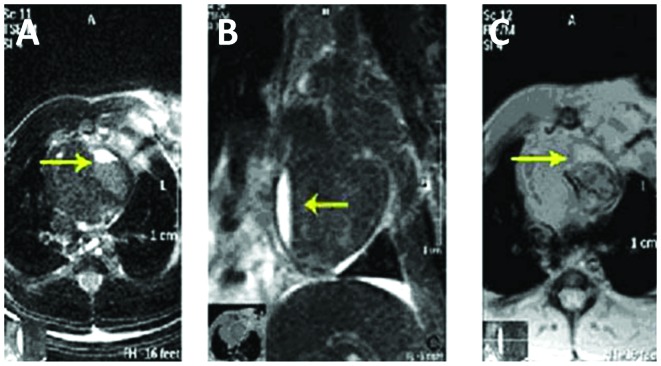
Magnetic resonance imaging of the infarcted region 2 weeks after coronary artery clamping. In the (A) transverse T2* image and (B) oblique sagittal T2* image, high signal intensity was observed in the area of the infarct. (C) In the transverse T2* image, the boundary between the ischemic region and the surrounding tissue was unclear.

**Figure 4:**
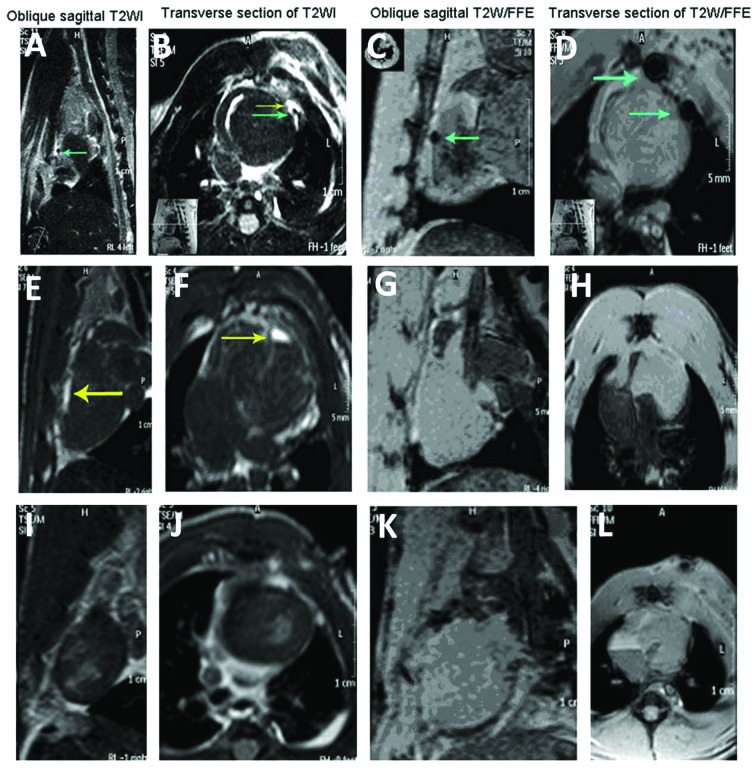
Magnetic resonance imaging of the myocardia of Sprague-Dawley rats 1 day post-transplantation of the BMSCs. (A–D) The superparamagnetic iron oxide-labeled BMSCs and the transplantation region had round, low intensity signals. (E–H) Unlabeled BMSCs. (I–L) Myocardium following phosphate-buffered saline injection. The green arrowheads indicate the region of transplanted BMSCs and the yellow arrowheads indicate the area of myocardial infarction. (A, E and I) are oblique sagittal sections and (B, F and J) are transverse sections from the T2 scans. (C, G and K) are oblique sagittal sections and (D, H and L) are transverse sections from the T2* scans. BMSCs, bone marrow mesenchymal stem cells; WI, weighted-imaging; FFE, fast field echo.

**Figure 5:**
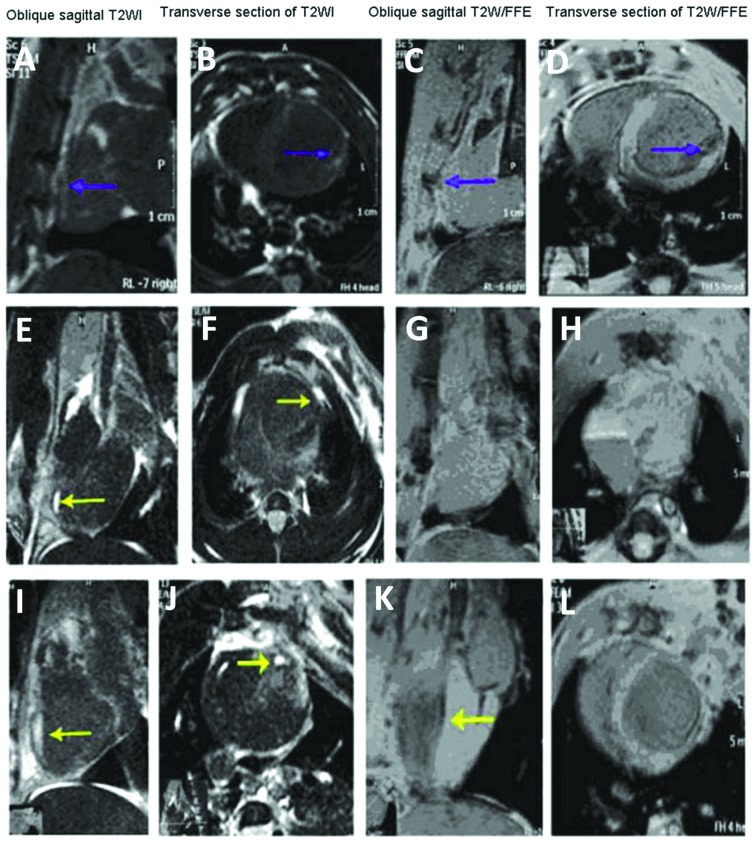
Magnetic resonance imaging of the myocardia of Sprague-Dawley rats 3 weeks post-transplantation of the BMSCs. (A–D) Superparamagnetic iron oxide-labeled BMSCs; low-intensity signals were observed. Compared with the images from day 1 post-transplantation, the transplantation region was enlarged and the signals were blurred. (E–H) Unlabeled BMSCs; the area of infarct decreased. (I–L) Left ventricular aneurysm formation was observed in the myocardium injected with phostphate-buffered saline. The blue arrowheads indicate the region of the transplanted BMSCs and the yellow arrowheads indicate the area of myocardial infarction.(A, E and I) are oblique sagittal sections from the T2 scans. Images (B, F and J) are transverse sections from the T2 scans. Images (C, G and K) are oblique sagittal sections from the T2* scans. Images (D, H and L) are transverse sections from the T2* scans. BMSCs, bone marrow mesenchymal stem cells.

The mean myocardial infarct size of the 60 rats was 1.71±0.13 cm^2^ and no difference was observed in the size of the infarct between the three groups. At 2 weeks following coronary artery ligation, a high-intensity signal was noted in the infarcted area on the T2 images (Fig. 3) and, on the first day following transplantation of the BMSCs, low-intensity MRI signals were found at the margins of the infarcted region on the T2 and T2* sequences (Fig. 4). The low-intensity signal decreased over time and became indistinct from the surrounding tissue. At 3 weeks, a low-intensity signal began to appear in the area of infarction; however, subsequently the signal intensity decreased and became indistinct (Fig. 5).

### SPIO-labeled BMSCs in the infarcted myocardium

**Figure 6:**
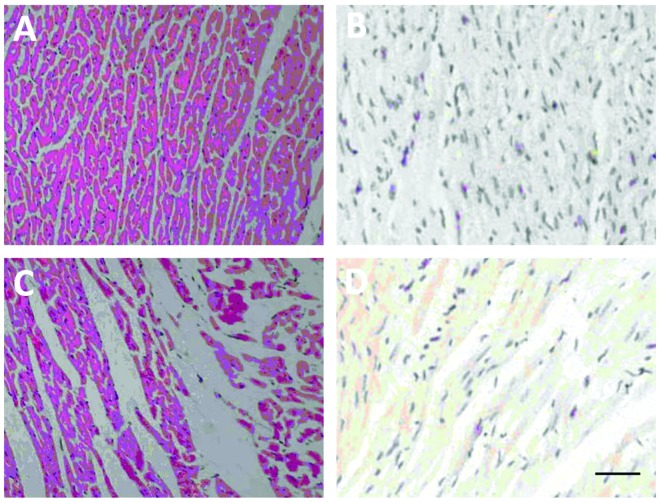
H&E and CD90 staining of the normal myocardia of Sprague-Dawley rats. In the normal myocardia, (A) normal morphology and regular arrangement of the myocardial cells was observed and (B) low levels or no expression of CD90 was observed in the normal myocardial tissue. The brown color indicates expression of CD90. In the myocardial tissue 2 weeks after myocardial infarction, (C) H&E staining revealed that myocardial fibers were irregularly arranged, nuclei were dissolved and a number of myocardial cells had disappeared in the myocardial ischemic region. (D) No expression of CD90 in the myocardial ischemic region was observed 2 weeks following myocardial infarction. The scale bar is 20 μm. H&E, hematoxylin and eosin; magnification, ×200.

**Figure 7:**
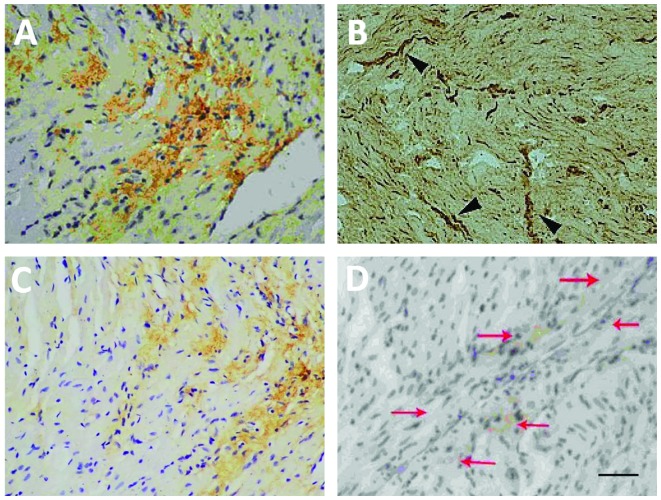
Hematoxylin and eosin and CD90 staining of the myocardia. One day after transplantation of the BMSCs, (A) marked expression of CD90 was observed in the SPIO-labeled BMSCs post-transplantation (brown region) and (B) unlabeled BMSCs revealed CD90 (+) staining. Arrows indicate CD90(+) staining cells. At 3 weeks post-transplantation of BMSCs, (C) SPIO-labeled BMSCs were CD90(+) and (D) BMSCs and myocardial fibers were arranged linearly. The red arrowheads indicate the transplanted region of the BMSCs. The scale bar is 20 μm. Magnification, ×200. BMSCs, bone marrow mesenchymal stem cells; SPIO, superparamagnetic iron oxide.

H&E staining revealed the presence of damaged myocardial cells in the infarcted region 2 weeks after coronary artery ligation (Fig. 6A and C). Immunohistochemical staining using the anti-CD90 antibody revealed low or no expression of CD90 in either the normal or infarcted myocardial tissues (Fig. 6B and D). The expression of CD90 was marked in the SPIO-labeled BMSCs 24 h after transplantation, suggesting successful transplantation of the BMSCs into the infarcted myocardium (Fig. 7A and B). The transplanted BMSCs remained undifferentiated 3 weeks after transplantation and were arranged along myocardial fibers in a strip-like manner (Fig. 7C and D).

## Discussion

In the present study, BMSCs were labeled with SPIO and MRI and *in vivo* tracking of the distribution and migration of BMSCs was performed 1 day and 3 weeks after BMSC transplantation. The results revealed that, from day 1 post-transplantation, a low-intensity signal was detected on T2* sequences. The intensity of the signal decreased with time; however, the area of the signal increased and gradually expanded into the infarcted myocardia. This suggests that *in vivo* tracking of SPIO-labeled BMSCs by MRI is feasible and effective. Notably, the signal intensity in the infarcted area further decreased and became indistinct 3 weeks post-transplantation, which was likely due to the effects of macrophages.

BMSCs are able to differentiate into myocardial cells in a suitable microenvironment. Previous studies investigating the effectiveness of stem cell transplantation in treating myocardial infarction have provided conflicting results. Tomita *et al* (22) first demonstrated that rat bone marrow stromal cells differentiate into cardiomyocytes *in vitro*, induce angiogenesis in ventricular scar tissue and ultimately improve myocardial function upon transplantation. In a study of 69 patients with acute myocardial infarction, intracoronary injection of autologous BMSCs led to significant improvement in the ejection fraction and the rate of motion of the infarcted region of the left ventricle (23). Analysis of data from the Autologous Stem Cell Transplantation in Acute Myocardial Infarction (ASTAMI) trial demonstrated that, although treatment with stem cells appeared to be safe in the long-term and was associated with significantly improved exercise time and heart rate response (24), further analysis of the data after 3 years revealed only a small improvement in exercise time (25). A 3year serial echocardiographic sub-study of the ASTAMI data indicated that improvement of the left ventricular ejection fraction and diastolic function as a result of acute percutaneous coronary intervention was not affected by the intracoronary injection of stem cells (26).

The mechanisms by which BMSCs may lead to the repair infarcted tissue remain to be elucidated. Possible mechanisms include the improvement of myocardial ischemia by the formation of new blood vessels and promotion of collateral circulation, an increase in the number of functional myocardial cells in the infarcted region, the promotion of host vascular proliferation and the improvement of cardiac function (27–30). The present study observed that SPIO-labeled BMSCs remained undifferentiated at the injection site for 3 weeks following transplantation.

Stem cells are similar to the surrounding tissue and magnetic contrast agents are necessary to alter the relaxation of BMSCs to enable detection by MRI. SPIO nanoparticles produce a hypointense footprint on MRI and, thus, exhibit a lower signal intensity on T2 images (12). In the present study, a significant difference was observed in the T2 images of the myocardium due to SPIO-labeled BMSCs, suggesting that SPIO labeling provides a measurable effect on MRI (5). In general, contrast agents are not effectively taken up by non-phagocytic cells; however, contrast agents are effectively incorporated into macrophages, which are abundant close to areas of infarction and migrate to the lesion core (31). Amsalem *et al* (18) demonstrated that, 4 weeks after the transplantation of MSCs, the transplanted cells were not present in the infarcted myocardium and enhanced MRI signals were observed that originated from cardiac macrophages that had engulfed the SPIO nanoparticles. In order to incorporate the contrast agents into stem cells, positively charged transfection agents, including polylysine and protamine sulfate, are often used to modify the surface electrical properties of the cells (32). Labeling stem cells using SPIO particles does not require a transfection agent and has proven to be useful for the *in vivo* detection of low signals in regions with transplanted cells (14).

The activity and reproductive capability of magnetically labeled cells determines their clinical application. The results of the present study indicated that SPIO nanoparticles at 25 μg/ml effectively labeled cells with no adverse effects on cell activity, growth or differentiation. Amsalem *et al* (18) also found that the SPIO labeling of stem cells did not affect their protective effect against progressive left ventricular dilatation and dysfunction is a rat model of myocardial infarction.

At present, the tracking of stem cells following transplantation in patients is conducted mainly using *in vitro* labeling and the three main imaging techniques used are labeling with radioisotopes, optical imaging and MRI, each of which has its own advantages and shortcomings (8). Several studies have examined the use of MRI/SPIO labeling to trace implanted stem cells (18,33–35). MRI has high temporal and spatial resolution, is non-invasive and does not use ionizing radiation. It can image any region of the body with high resolution and provides a long time-window for imaging. In addition, MRI can simultaneously obtain physiological, molecular and anatomical information and enables observation of the dynamic process of cell migration. However, the technique suffers from low sensitivity requiring an increased number of cells for successful tracking (14).

There are a number of limitations of the present study that must be considered. No Prussian blue staining was used to colocalize the SPIOs and CD90^+^ cells. In addition, CD90 was used as a marker of the grafted cells; however, green fluorescent protein or other membrane labeling, such as using dialkylcarbocyanines, is preferable (36). The regions of interest were not normalized between scans, cardiac function was not assessed and no quantitative analysis of the signal effects of SPIO in the infarcted and non-infarcted myocardium was performed. The limitations of the present study limit the ability to state with 100% certainty that the SPIO was harbored in the stem cells and not the macrophages; however, the data and temporal findings indicate that the SPIO was indeed in the stem cells.

Therefore, BMSCs can be readily labeled with SPIO, without using a transfection agent, using a simple and effective labeling method. MRI scans can then be used for *in vivo* tracking of the distribution of the SPIO-labeled BMSCs.

## Figures and Tables

**Table I:** Cell viability of SPIO-labeled and unlabeled bone marrow mesenchymal stem cells.

Time	SPIO-labeled (n=10)	Unlabeled (n=10)	P-value^a^
6 h	98.65±1.63	98.74±1.42	0.897
12 h	96.35±0.96	96.42±1.33	0.894
24 h	95.51±1.44	95.78±1.21	0.655

Data are presented as the mean ± standard deviation.

a Independent t-test.

SPIO, superparamagnetic iron oxide.
